# Tryptophan metabolism induced by TDO2 promotes prostatic cancer chemotherapy resistance in a AhR/c-Myc dependent manner

**DOI:** 10.1186/s12885-021-08855-9

**Published:** 2021-10-17

**Authors:** Fan Li, Zhenyu Zhao, Zongbiao Zhang, Yan Zhang, Wei Guan

**Affiliations:** Department of Urology, Tongji Hospital, Tongji Medical College, Huazhong University of Science and Technology, Wuhan, China

**Keywords:** Tryptophan metabolism, Kynurenine, AhR, C-Myc, Prostatic cancer

## Abstract

**Background:**

Tumor cells exhibit enhanced metabolism of nutrients to satisfy the demand of sustained proliferation in vivo. Seminal reports have presented evidence that tryptophan (Trp) metabolic reprogramming induced by aberrant indoleamine 2,3-dioxygenases could promote tumor development in several cancer types. However, the underlying mechanism of Trp metabolism associated tumor progression is not fully understood.

**Materials and methods:**

Prostatic cell lines LNCaP and VCaP were purchased from the Cell Bank of the Chinese Academy of Sciences (China). Human prostatic tumor tissue samples were obtained from the Tongji Hospital. Female NOD-SCID mice (6 ~ 8 weeks) were purchased from Huafukang Co. (China) and raised in SPF room. Commercial kits and instruments were used for cell apoptosis analysis, real-time PCR, western blotting, ELISA analysis and other experiments.

**Result:**

Comparing the tumor tissues from prostatic cancer patients, we found elevated expression of tryptophan 2, 3-dioxygenase 2 (TDO2), and elevated Trp metabolism in chemo-resistant tumor tissues. In vitro, overexpression of TDO2 significantly promoted the Trp metabolism in prostatic cancer cell lines LNCaP and VCap, resulting in the multidrug resistance development. Mechanistically, we demonstrated that Trp metabolite kynurenine (Kyn) promoted the upregulation and nuclear translocation of transcription factor aryl hydrocarbon receptor (AhR). Subsequently, AhR collaborated with NF-κB to facilitate the activation of c-Myc. In turn, c-Myc promoted the up-regulation of ATP-binding cassette (ABC) transporters and Trp transporters, thereby contributing to chemoresistance and strengthened Trp metabolism in prostatic cancer. Interrupt of Trp/TDO2/Kyn/AhR/c-Myc loop with c-Myc inhibitor Mycro-3 efficiently suppressed the chemoresistance and improved the outcome of chemotherapy, which described a new strategy in clinical prostatic cancer treatment.

**Conclusion:**

Our study demonstrates that elevated TOD2 expression promoted Trp metabolism and metabolite Kyn production, thus resulting in the activation of AhR/c-Myc/ABC-SLC transporters signaling pathway. Interrupt of Trp metabolism/c-Myc loop efficiently suppressed the drugs resistance induced by TDO2, which represented potential target to improve the outcome in drug-resistant prostatic cancer treatment.

**Supplementary Information:**

The online version contains supplementary material available at 10.1186/s12885-021-08855-9.

## Introduction

Prostatic cancer is the most commonly diagnosed cancer, with the third leading cause of cancer associated death in males [1]. The high mortality rates (> 10 per 10,000 people) were found in many parts of regions, including Central America, Australia, New Zealand and Western Europe [2]. In many cases, successful treatment of prostatic cancer is difficult due to the late detection and rate of metastasis [3]. Importantly, the major cause of prostatic cancer associated death is the development of chemoresistance [4]. Therefore, clarifying the mechanism of multidrug resistance and selecting appropriate treatment are critical for improving outcomes of prostatic cancer treatment.

The development of chemoresistance in cancer cells is determined by diverse factors, including the presence of cancer stem cells, DNA damages repair, tumor microenvironment, and aberrant metabolism [5, 6]. The reprogramming of tumor metabolism is tightly associated with the adaptability of tumor cells after chemotherapy. In fact, the reprogramming of metabolism further affects the principal metabolic pathways, which eventually gives rise to cell flexibility and allows tumor cells to escape chemotherapy [7]. Among various amino acids, Trp served as important element for protein synthesis, and the metabolite of Trp is proved to meditate the activation of Kyn associated pathway [8]. Altered Trp metabolism and increased production of Trp metabolites in solid tumors, including breast, colon, and bladder cancer [9–11] have been known as crucial driver of cancer development. Among Trp metabolites, Kyn accounts for approximately 95% production of ingested Trp, not used for serotonin pathway or protein synthesis [12]. In Trp metabolism, Trp is oxidized by indoleamine 2,3-dioxygenase-1/2 (IDO1/2) or TDO2, and produce formylkynurenine, which is further degraded to Kyn [13]. The overexpression of IDO1 has been observed in many tumor types. And both tumor and stromal cells have been proved to exhibit elevated IDO1 activity [14, 15]. Intriguingly, current findings suggested that the expression TDO, rather than IDO, might play an essential role in promoting tumor development [16]. However, the significance of Trp metabolic enzymes IDO and TDO activity in drugs resistance remains controversial and has yet to be explored.

Recent advances in understanding of Trp metabolism, including its regulatory functions and associated molecular targets, have expanded the opportunity to target Trp metabolic pathways for improved oncotherapy regimens. Compelling literatures have reported redundant pathways that caused constitutive IDO1 expression and activation in tumor cells. Diverse pro-inflammatory signals, including IFN-γ, CpG DNA and lipopolysaccharide, have been demonstrated to promote the expression of IDO1 [17–19]. Meanwhile, Wnt5α was also reported to mediate IDO1 activity via a β-catenin dependent signaling pathway in dendritic cells [20], and facilitate the IDOs activation through the AhR-IL-6-STAT3 signaling loop in several tumor types [21]. In contrast to IDO1, the regulation of TDO2 in tumors remains poorly understood. Diverse mechanisms have been proved to participate in the regulation of TDO, such as tryptophan and cofactor associated signaling activation, heme/hormonal induced TDO expression [22, 23]. Increasing evidence also suggested that TDO could be upregulated by glucagon and estrogen [24]. The activation of AhR signals induced by Kyn and TDO2 have been observed in lung cancer and demonstrated to be involved in the survival and motility of cancer cells [25]. Despite of this, the exact mechanism by which Trp metabolism mediates multidrug resistance in prostatic cancer remains exclusive.

Herein, we further discovered the role of Trp metabolism in prostatic cancer, and TDO2/Kyn axis was responsible for this process. Mechanistically, our research firstly demonstrated that Trp metabolite Kyn promoted AhR/c-Myc/ABC transporters activation to mediate the drugs. In turn, c-Myc up-regulated the expression of Trp transporters, which further promoted Trp metabolism in cancer cells. Interrupt of Trp metabolism/c-Myc loop efficiently reversed the chemo-resistance induced by TDO2, which open novel therapeutic avenues for prostatic cancer treatment.

## Materials and methods

### Cell lines and reagents

Prostatic cell lines LNCaP and VCaP were purchased from the Cell Bank of the Chinese Academy of Sciences (China). LNCaP cells were maintained in complete Roswell Park Memorial Institute 1640 culture medium (Gibco, USA) containing 10% fetal bovine serum (Gibco, USA) at 37 °C. VCaP cells were maintained in Dulbecco’s modified Eagle’s medium (Gibco, USA) containing 10% fetal bovine serum (Gibco, USA) at 37 °C. NF-kB inhibitor pyrrolidinedithiocarbamate ammonium (PA), c-Myc inhibitor Mycro 3 were purchased from MCM (USA). Docetaxel (Doc), Abiraterone (Abi) and doxorubicin (Dox) were purchased from Sigma (USA). Other chemical reagents or materials were of high-performance liquid chromatography grade and obtained from Biyuntian (China).

### Tumor tissues collection

Human prostatic tumor tissue samples were obtained from the Tongji Hospital. The tumor tissues were collected in 10% formalin after surgical operation. Patients were divided into the chemo-sensitive (C-S) and chemo-resistant (C-R) groups according to follow-up visit. The protocols were approved by the ethics board of the Tongji Hospital. Written informed consent was obtained from patients before clinical experiments, and all protocols were designed in accordance with the Declaration of Helsinki.

### Cell apoptosis analysis

The cytotoxicity of LNCaP and VCaP cells to agents was determined by the FITC-Annexin V/ PE-PI apoptosis detection kit (Becton and Dickinson Co., USA). Briefly, agents treated tumor cells were resuspended and stained with Annexin V/PI staining solution for 15 min at room temperature. Then cells apoptosis was analyzed on a C6 flow cytometer (Becton and Dickinson Co., USA). Each experiment was repeated for three independent times.

### Real-time PCR

The targeted genes expression of LNCaP and VCaP cells were examined using quantitative real-time PCR. 1μg cDNA was used as template for amplification with SYBRÔ Green Real-Time PCR master mixes (Thermo Fisher Scientific, MA, USA). The primer pairs were used as follow: human ABCB1 forward primer 5′-TTGGCTGATGTTTGTGGGAAG-3′, and reverse primer 5′-CCAAAAATGAGTAGCACGCCT-3′; human ABCB2 forward primer 5′-TGCCCCGCATATTCTCCCT-3′, and reverse primer 5′-CACCTGCGTTTTCGCTCTTG-3′; human ABCG2 forward primer 5′-CAGGTGGAGGCAAATCTTCGT’, and reverse primer 5′-ACCCTGTTAATCCGTTCGTTTT-3′; human ABCC1 forward primer 5′-CTCTATCTCTCCCGACATGACC-3′, and reverse primer 5′-CTGAAGACTGAACTCCCTTCCT-3′; human ABCC3 forward primer 5′-TGGGGTGAAGTTTCGTACTGG-3′, and reverse primer 5′-CACGTTTGACTGAGTTGGTGATA-3′; human ABCC5 forward primer 5′-AGTCCTGGGTATAGAAGTGTGAG-3′, and reverse primer 5′-ATTCCAACGGTCGAGTTCTCC-3′; human ABCC6 forward primer 5′-AAGGAGGTACTAGGTGGGCTT-3′, and reverse primer 5′-CCAGTAGGACCCTTCGAGC-3′; human SLC16A1 forward primer 5′-AGGTCCAGTTGGATACACCCC-3′, and reverse primer 5′-GCATAAGAGAAGCCGATGGAAAT-3′; human SLC22A3 forward primer 5′-ATCGTCAGCGAGTTTGACCTT-3′, and reverse primer 5′-ACCTGTCTGCTGCATAGCCTA-3′; human SLC22A1 forward primer 5′-ACGGTGGCGATCATGTACC-3′, and reverse primer 5′-CCCATTCTTTTGAGCGATGTGG-3′; human SLCEA5 forward primer 5′-GAGCTGCTTATCCGCTTCTTC-3′, and reverse primer 5′-GGGGCGTACCACATGATCC-3′; human SLC7A8 forward primer 5′-AGGCTGGAACTTTCTGAATTACG-3′, and reverse primer 5′-ACATAAGCGACATTGGCAAAGA-3′; human SLC16AE forward primer 5′-CGTGGAGGCTTCTCTCACAG-3′, and reverse primer 5′-CGTAGGACAGCCCGTTTATCG-3′; human GAPDH forward primer 5′-GGAGCGAGATCCCTCCAAAAT-3′, and reverse primer 5′-GGCTGTTGTCATACTTCTCATGG-3′; the GAPDH was set as a control. Each experiment was performed in triple.

### Western blotting

LNCaP and VCaP cells were lysed using radioimmunoprecipitation assay buffer (Beyotime, China). Protease inhibitors (Beyotime, China) was added into the lyse buffer to avoid protein degradation. Samples (20 μg) were separated using sodium dodecyl sulfate polyacrylamide gel electrophoresis. Then samples were transferred onto nitrocellulose membranes. After that, samples were incubated with following primary antibodies: anti-AhR (1:500, Abcam, UK), anti-P-gp (1:1000, Abcam, UK), anti-β-actin (1:1000, Abcam, UK) and anti-c-Myc (1800, Abcam, UK). chemiluminescence kit (Beyotime, China) were used for protein detection. Each experiment was repeated for three independent times.

### Gene interference

For TDO2 and c-Myc overexpression, cDNAs for human TDO2 and c-Myc were synthesized by Ruibo Inc., China, The TDO2 or c-Myc cDNAs were inserted into pLVX-EF1α-IRES-Puro lentiviral vector (Takara, Japan) for stable overexpression in LNCaP and VCaP cells. The overexpression of TDO2 or c-Myc was examined by western blotting.

### Immunohistochemical and immunofluorescence

Pathological sections of prostatic tissues were retrieved using microwave antigen retrieval (Thermo, USA). Then samples were blocked by 5% Bovine Serum Albumin, followed by incubating with anti-TDO2 antibody (1:200, abcam, UK), anti-Kyn antibody (1:300, abcam, UK), anti-AhR antibody (1:200, abcam, UK), anti- NF-κB (1:200, abcam, UK) and anti-c-Myc antibody (1:500, abcam, UK) for 4 °C overnight. For immunofluorescence staining, the sections were then incubated with secondary antibodies (1:1000; abcam, UK), and the nucleus was stained with DAPI. Images were captured using a FV1000 laser scanning confocal microscope (Leica, Germany). For immunohistochemical staining, the sections were then stained using the ABC HRP Kit (Thermo, USA) and counterstaining with hematoxylin. Immunohistochemical images were captured using microscope (Leica, Germany). The intensity of proteins in sections were analyzed by Image-Pro Plus 2.0 software.

### Elisa analysis

The human Kyn Elisa kit was purchased from Keshun (China). The human Trp Elisa kit was purchased from BIOMAT (USA). For Kyn or Trp analysis, 10^5^ tumor cells were cultured in 2 ml medium at 37 °C. After 0 and 48 h, the supernatant was collected for Kyn or Trp concentration analysis according to the guidance of Kit. Each experiment was performed in triple independently.

### Animal protocols

Female NOD-SCID mice (6 ~ 8 weeks) were purchased from Huafukang Co. (China) and raised in SPF room. All experiments and protocols were approved and monitored by the Animal Care and Use Committee of Tongji Hospital. For tumor suppressive effects, LNCaP cells (2 × 10^6^ cells in 50 μl of PBS) were subcutaneously injected into NOD-SCID mice. After two weeks, the mice were treated with PBS, Doc (5 mg/kg), Abi (10 mg/kg), Mycro-3 (5 mg/kg) or combination every three days. The tumor sizes (*n* = 6 per group) and survival times (n = 6 per group) of the mice were recorded every day. The tumor volume was calculated according the formula: length × width^2^ × 0.5.

### Statistical analysis

The survival of patients from TCGA database was downloaded from https://www.cbioportal.org/. Each experiment was performed in triplicate independently. Data are expressed as the mean ± standard deviation. Differences among groups were determined suing variance or Student’s t-test analysis by GraphPad Software (USA) and SPSS software (USA). Survival times were analyzed by a log-rank test. * means *p* < 0.05, ** means *p* < 0.01, and n.s means no significant difference.

## Results

### TOD2 expression promoted Trp metabolism to facilitate drugs resistance in prostatic cancer

**Fig. 1:**
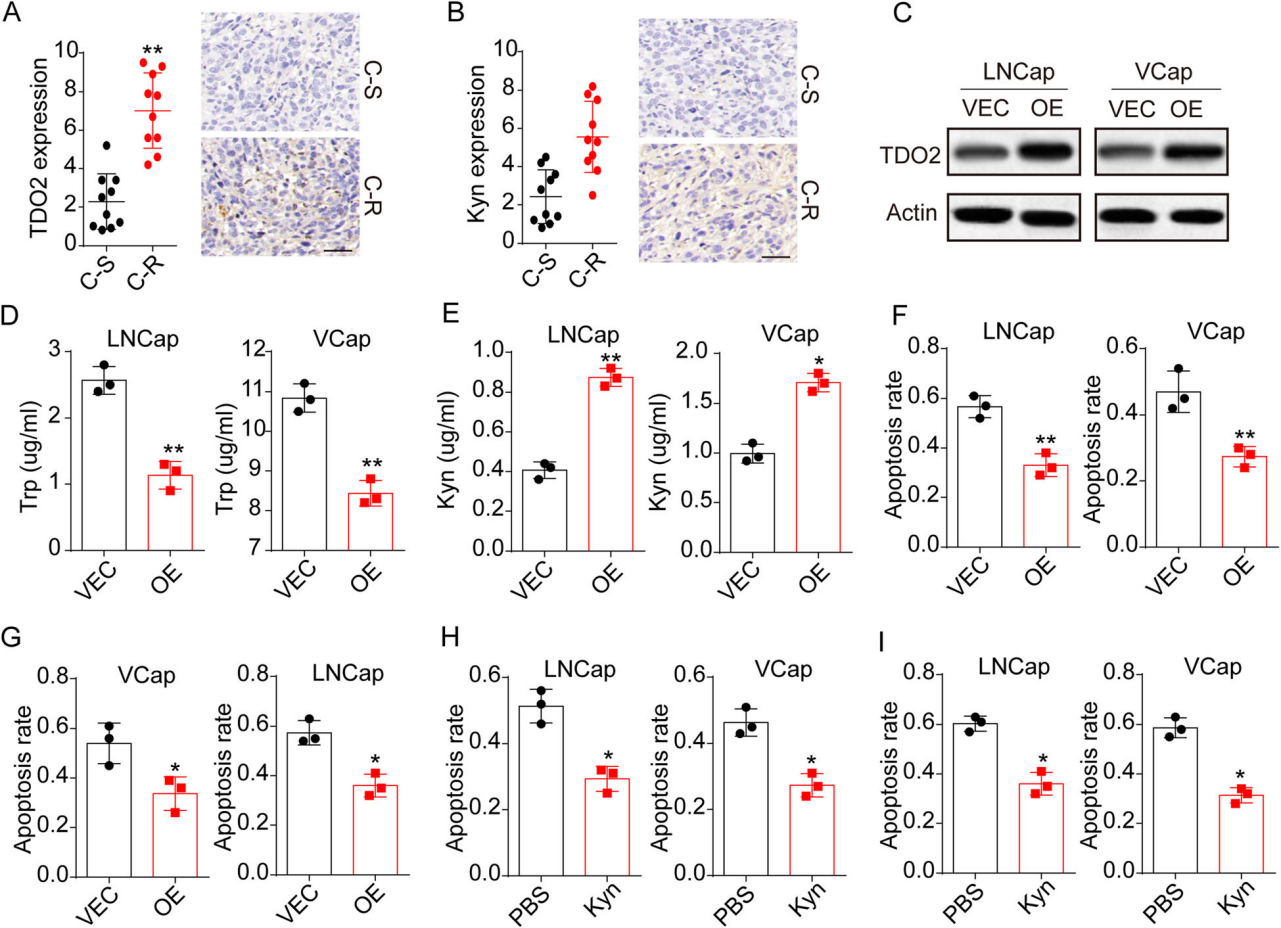
elevated Trp metabolism correlated with drugs resistance in prostate cancer. A, immunohistochemical staining of TDO2 in prostatic tumor tissues from chemo-sensitive (C-S) and chemo-resistant (C-R) patients (*n* = 10 per groups). B, immunohistochemical staining of Kyn in prostatic tumor tissues from chemo-sensitive (C-S) and chemo-resistant (C-R) patients (n = 10 per groups). C, western blotting of TDO2 in LNCaP/VCaP cells (VEC) and TDO2 overexpression LNCaP/VCaP cells (OE). D, Trp concentration in culture medium of LNCaP/VCaP cells (VEC) and TDO2 overexpression LNCaP/VCaP cells (OE). 10^5^ cells were cultured in 2 ml culture medium for 48 h. E, Kyn concentration in culture medium of LNCaP/VCaP cells (VEC) and TDO2 overexpression LNCaP/VCaP cells (OE). 10^5^ cells were cultured in 2 ml culture medium for 48 h. F, cell apoptosis of LNCaP/VCaP cells (VEC) and TDO2 overexpression LNCaP/VCaP cells (OE) treated with Abi (10 μM). G, cell apoptosis of LNCaP/VCaP cells (VEC) and TDO2 overexpression LNCaP/VCaP cells (OE) treated with Doc (1 μg/ml). H, cell apoptosis of LNCaP/VCaP cells (PBS or 200 μM Kyn cultured) treated with Abi (10 μM). I, cell apoptosis of LNCaP/VCaP cells (PBS or 200 μM Kyn cultured) treated with Doc (1 μg/ml)

To determine the potential role of Trp metabolism in drugs resistance development, we collected tumor tissues from patients with prostatic cancer. The tumor tissues were divided into C-S and C-R groups according to the follow-up visit after surgery. Intriguingly, increased tryptophan 2,3-dioxygenase TDO2 was observed in C-R tumor tissues (Fig. 1A), thus indicating enhanced Trp metabolism in C-R prostatic tumor tissues. To further confirm our hypothesis, we compared the Trp metabolite Kyn expression in C-S and C-R tumor tissues from patients. And we found that Kyn secretion was obviously upregulated in C-R tumor tissues (Fig. 1B), suggesting that TDO2 promoted Trp metabolite Kyn production in C-R prostatic cancer cells. Subsequently, we extended our studies to evaluate the role of Trp metabolism in prostatic cancer cell lines LNCaP and VCaP. First, we overexpressed TDO2 in LNCaP and VCaP cells (Fig. 1C) and examined the Trp/Kyn metabolism. Consistent to our hypothesis, elevated Trp uptake and Kyn production occurred in TDO2 overexpression LNCaP and VCaP cells (Fig. 1D and E). To further identify the role of Trp metabolism, we detected the cytotoxicity of Abi and Doc to TDO2 overexpression prostatic cancer cells. LNCaP and VCaP cells with elevated TOD2 expression revealed obvious Abi (Fig. 1F) and Doc (Fig. 1G) resistance compared with control group. Similarly, Kyn treatment promoted the Abi (Fig. 1H) and Doc (Fig. 1I), indicating that TDO2 associated Trp metabolism promoted drugs resistance via the metabolite Kyn. Together, those results reminded that aberrant TDO2 expression and Trp metabolism facilitated prostatic cancer drugs resistance through a Kyn dependent manner.

### Kyn promoted AhR activation to mediate drugs resistance

**Fig. 2:**
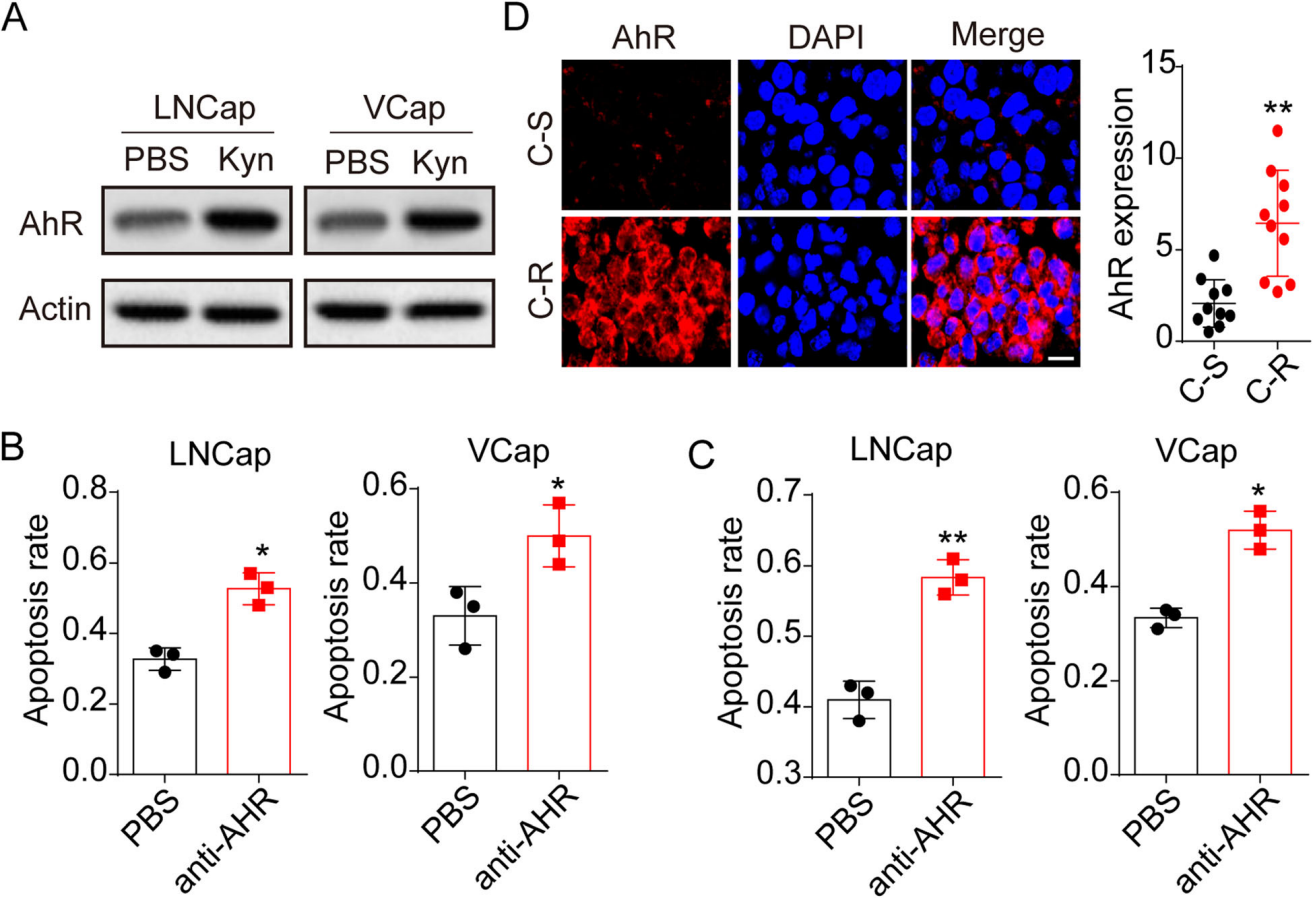
Kyn promoted the activation of AhR signals to mediate drugs resistance. A, western blotting of AhR in LNCaP/VCaP cells treated with PBS or 200 μM Kyn. B, cytotoxicity of Kyn (200 μM) cultured LNCaP/VCaP cells treated with PBS or AhR antagonist 2 (2 nM) to Abi (10 μM). C, cytotoxicity of Kyn (200 μM) cultured LNCaP/VCaP cells treated with PBS or AhR antagonist 2 (2 nM) to Doc (1 μg/ml). D, immunofluorescence staining of AhR in prostatic tumor tissues from chemo-sensitive (C-S) and chemo-resistant (C-R) patients (n = 10 per groups)

Next, we sought to interrogate the mechanism by which Kyn promoted prostatic cancer drugs resistance. Kyn derived from tumor cells has been shown previously to mediate the activation of AhR signals, thus contributing to immunosuppressive effects in T cells. Here, increased expression of AhR was observed in Kyn treated prostatic cancer cells (Fig. 2A), suggesting that AhR might be involved in the Kyn associated chemoresistance. To further determine the role of AhR, we employed AhR antagonist 2 to block the AhR signals in LNCap and VCap cells. To avoid the cell apoptosis induced by AhR inhibition, we examined the cell apoptosis in LNCaP/VCaP cells treated with AhR antagonist 2 or AhR knockout cells. No obvious cell apoptosis was observed in LNCaP/VCaP cells when AhR inhibition (Fig. S1A and B). However, LNCaP and VCaP cells, treated with Kyn, revealed weakened Abi (Fig. 2B) and Doc (Fig. 2C) resistance in the presence of AhR antagonist 2. More importantly, enhanced expression of AhR was found in tumor tissues from C-R patients with prostatic cancer (Fig. 2D), indicating that Kyn promoted AhR activation to mediate drugs resistance in prostatic cancer.

### AhR collaborated with NF-κB to facilitate the activation of c-Myc

**Fig. 3:**
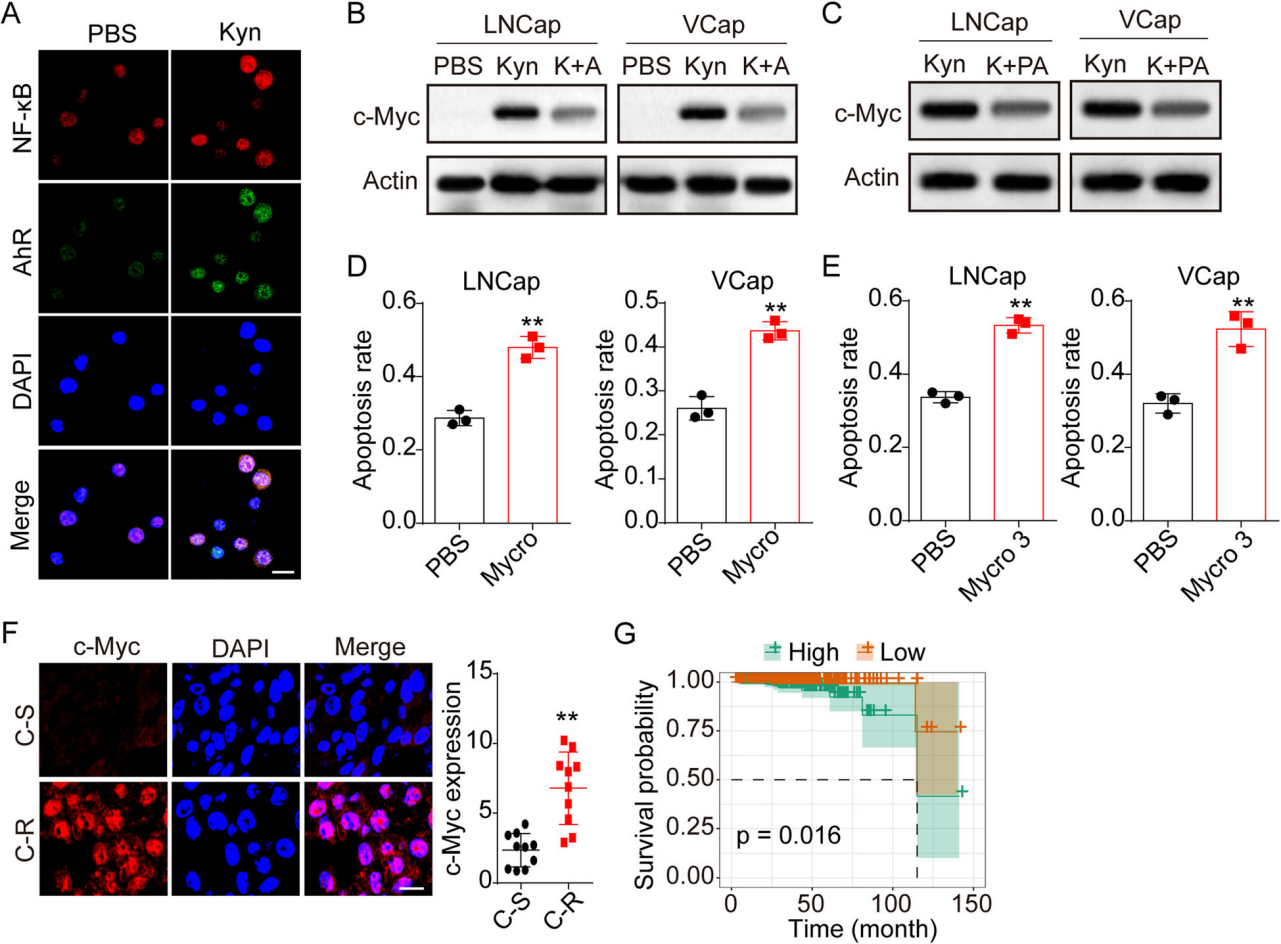
AhR collaborated with NF-κB to facilitate the activation of c-Myc. A, immunofluorescence staining of AhR and NF-κB in LNCaP cells treated with PBS or Kyn (200 μM). The scale bar was 15 μm. B, western blotting of c-Myc in LNCaP/VCaP cells treated with PBS, Kyn (200 μM) and Kyn (200 μM) combing AhR antagonist 2 (2 nM). C, western blotting of c-Myc in LNCaP/VCaP cells treated with Kyn (200 μM) and Kyn (200 μM) combing PA (10 μM). D, cytotoxicity of Kyn (200 μM) cultured LNCaP/VCaP cells treated with PBS or Mycro 3 (1 μM) to Abi (10 μM). E, cytotoxicity of Kyn (200 μM) cultured LNCaP/VCaP cells treated with PBS or Mycro 3 (1 μM) to Doc (1 μg/ml). F, immunofluorescence staining of c-Myc in prostatic tumor tissues from chemo-sensitive (C-S) and chemo-resistant (C-R) patients (n = 10 per groups). The scale bar was 15 μm. G, the overall survival of patients with prostate cancer in high c-Myc expression and low c-Myc expression (*n* = 275)

AhR is strictly associated with the pro-survival signals activation in cancer cells, and believed to collaborate with NF-κB to induce the c-Myc expression in mammalian cells. Indeed, colocalization of AhR and NF-κB was observed in Kyn treated LNCaP cells (Fig. 3A). Meanwhile, elevated expression of c-Myc was found in Kyn treated tumor cells, and suppression of AhR inhibited the expression of c-Myc (Fig. 3B). Additionally, using NF-κB inhibitor PA, we found that suppression of NF-κB contributed to the downregulation of c-Myc (Fig. 3C), suggesting that the c-Myc activation induced by Kyn/AhR signal was NF-κB dependent. Subsequently, we sought to determine whether c-Myc expression participated in the chemoresistance development in prostatic cancer. To avoid the cell apoptosis induced by c-Myc inhibition, we examined the cell apoptosis in LNCaP/VCaP cells treated with c-Myc inhibitor Mycro-3 or c-Myc knockout cells. No obvious cell apoptosis was observed in LNCaP/VCaP cells when c-Myc inhibition (Fig. S1C and D). We treated LNCaP and VCaP with c-Myc inhibitor Mycro 3 and Kyn, following with chemotherapy cytotoxicity analysis. Intriguingly, LNCaP and VCaP pre-treated with Mycro-3 displayed weakened Abi/Doc resistance compared to the control group (Fig. 3D and E). We also probed the expression of c-Myc in our clinical tumor samples and evaluated the overall survival in c-Myc high expression prostatic cancer patients (TCGA database). As anticipated, tumor tissues from C-R prostatic cancer patients revealed enhanced c-Myc expression (Fig. 3F), and poor overall survival was observed in c-Myc high expression prostatic cancer patients (Fig. 3G). Those results implicated that AhR collaborated with NF-κB to facilitate the activation of c-Myc, thereby promoting the drugs resistance of prostatic cancer.

### C-Myc up-regulated the expression of Trp transporters and ABC transporters

**Fig. 4:**
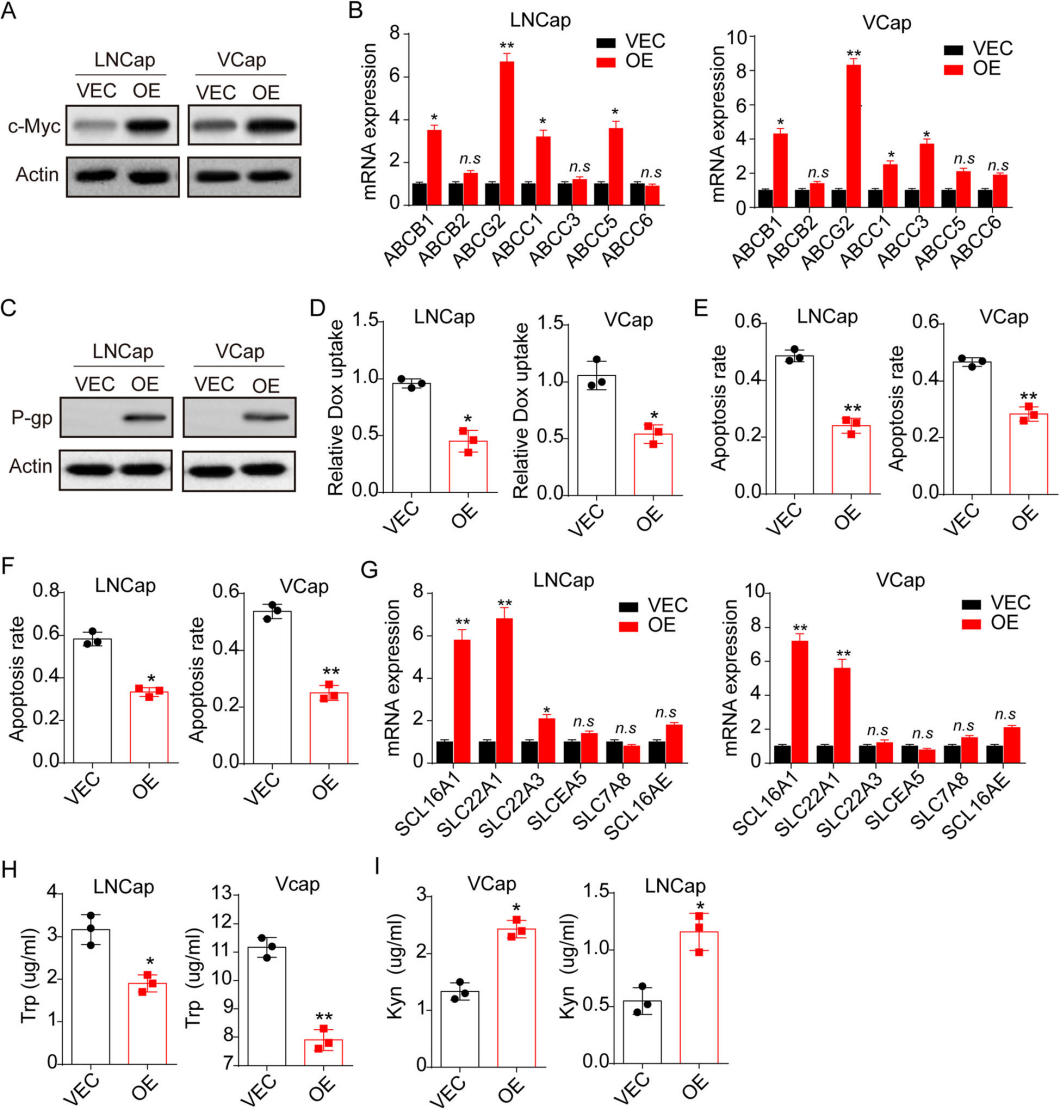
c-Myc up-regulated the expression of Trp transporters and ABC transporters. A, western blotting of c-Myc in LNCaP/VCaP cells (VEC) and c-Myc overexpression LNCaP/VCaP cells (OE). B, the expression of ABCB1, ABCB2, ABCG2, ABCC1, ABCC3, ABCC5, ABCC6 in LNCaP/VCaP cells (VEC) and c-Myc overexpression LNCaP/VCaP cells (OE) detected by real-time PCR. C, the western blotting of P-gp in LNCaP/VCaP cells (VEC) and c-Myc overexpression LNCaP/VCaP cells (OE). D, relative uptake of Dox in LNCaP/VCaP cells (VEC) and c-Myc overexpression LNCaP/VCaP cells (OE). E, cell apoptosis of LNCaP/VCaP cells (VEC) and c-Myc overexpression LNCaP/VCaP cells (OE) treated with Abi (10 μM). F, cell apoptosis of LNCaP/VCaP cells (VEC) and c-Myc overexpression LNCaP/VCaP cells (OE) treated with Doc (1 μg/ml). G, the expression of SLC16A1, SLC22A1, SLC22A3, SLCEA5, SLC7A8, SLC16AE in LNCaP/VCaP cells (VEC) and c-Myc overexpression LNCaP/VCaP cells (OE) detected by real-time PCR. H, Trp concentration in culture medium of LNCaP/VCaP cells (VEC) and c-Myc overexpression LNCaP/VCaP cells (OE). 10^5^ cells were cultured in 2 ml culture medium for 48 h. I, Kyn concentration in culture medium of LNCaP/VCaP cells (VEC) and c-Myc overexpression LNCaP/VCaP cells (OE). 10^5^ cells were cultured in 2 ml culture medium for 48 h

The c-Myc oncogene is transcriptional regulator that triggers tumorigenesis through the transcriptional modulation of many genes, including multidrug resistance associated ABC transporters. To determine whether c-Myc facilitated prostatic cancer drugs resistance through a multidrug resistant protein associated manner, we overexpressed c-Myc in LNCaP/VCaP cells (Fig. 4A) and examined the expression of major ABC transporters using q-PCR. Intriguingly, elevated expression of ABCB1, ABCG2 and ABCC1 were observed in c-Myc overexpression prostatic cells (Fig. 4B). Consistently, multidrug resistant protein P-glycoprotein (P-gp), which is encoded by ABC genes, was upregulated in c-Myc overexpression cells (Fig. 4C), indicating that c-Myc promoted multidrug resistant protein P-gp in prostatic cancer. Next, we sought to examine the drugs uptake in c-Myc overexpression prostatic cancer cells, and doxorubicin was employed as the model drug, which could be detected by flow cytometry. Reduced doxorubicin was taken up in c-Myc overexpression cells, when compared to the control group (Fig. 4D), which was consistent to out hypothesis that c-Myc upregulated ABC transporters to suppressed drugs uptake. Undoubtedly, c-Myc overexpression LNCaP and VCaP cells displayed enhanced Abi (Fig. 4E) and Doc (Fig. 4F) resistance. Increasing evidence has presented that Trp transporters could be upregulated in tumor cells with aberrant Trp metabolism. To confirm whether Trp transporters were upregulated by c-Myc, we further performed q-PCR to examine the major Trp transporters SLC16A1, SLC22A1, SLC22A3, SLCEA5, SLC7A8, SLC16AE in LNCaP/VCaP cells and c-Myc overexpression LNCaP/VCaP cells. Indeed, c-Myc obviously promoted the expression of SLC16A1 and SLC22A11 (Fig. 4G), and c-Myc overexpression LNCaP/VCaP cells displayed enhanced Trp uptake (Fig. 4H) and Kyn production (Fig. 4I), suggesting that c-Myc strengthened the Trp transporters, which further facilitated the Trp metabolism induced by TDO2. Together, these results demonstrated the c-Myc expression promoted ABC transporters to mediate drugs resistance, and upregulated SLC transporters to establish a Trp/TDO2/Kyn/AhR/c-Myc/SLC transporters metabolic loop.

### Interrupt of Trp metabolism/c-Myc loop by Mycro-3 improved the outcome of chemotherapy

**Fig. 5:**
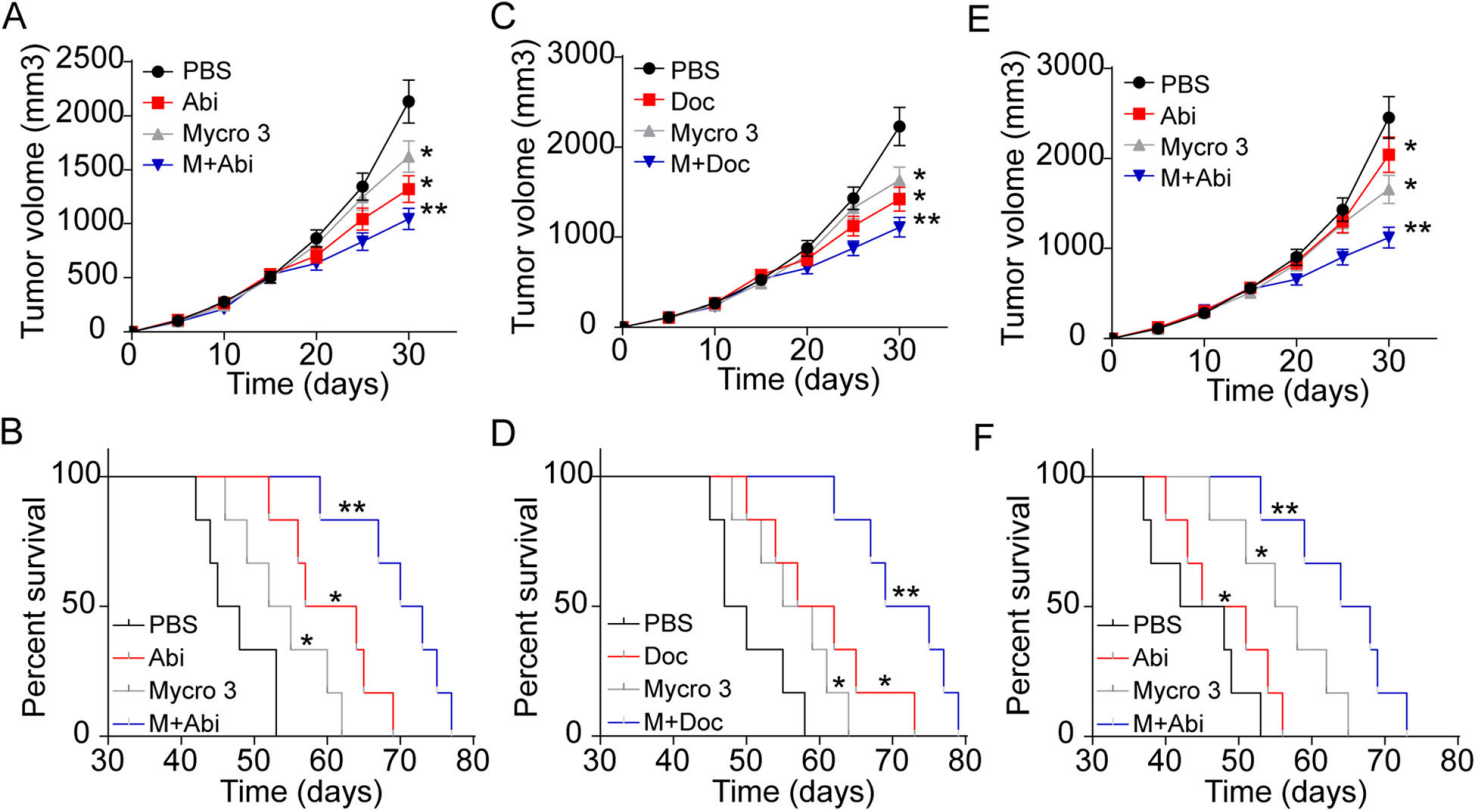
Interrupt of Trp metabolism/c-Myc loop improved the outcome of chemotherapy in prostate cancer. A, tumor volumes of LNCaP bearing mice treated with PBS, Abi, Mycro 3 and Abi combing Mycro 3. B, survival time of LNCaP bearing mice treated with PBS, Abi, Mycro 3 and Abi combing Mycro 3. C, tumor volumes of LNCaP bearing mice treated with PBS, Doc, Mycro 3 and Doc combing Mycro 3. D, survival time of LNCaP bearing mice treated with PBS, Doc, Mycro 3 and Doc combing Mycro 3. E, tumor volumes of c-Myc overexpression LNCaP bearing mice treated with PBS, Abi, Mycro 3 and Abi combing Mycro 3. F, survival time of c-Myc overexpression LNCaP bearing mice treated with PBS, Abi, Mycro 3 and Abi combing Mycro 3. G, schematic diagram of Trp metabolism/c-Myc induced drugs resistance in prostatic cancer

Given the essential role of c-Myc in Trp metabolism loop, interrupt Trp metabolism/c-Myc loop by Mycro-3 might be feasible to reverse the drug resistance in prostatic cancer. We used LNCaP cells to establish a subcutaneous LNCaP prostatic cancer mice model for anticancer treatment analysis. Mycro-3 combining Abi obviously inhibited the LNCaP prostatic cancer development and prolonged the overall survival of mice (Fig. 5A and B). Similar anticancer effects were found in tumor bearing mice treated with Mycro-3 combining Doc (Fig. 5C and D). Next, we sought to evaluate the anticancer effects of Mycro-3 in chemo-resistant prostatic cancer model. Here, we establish a subcutaneous prostatic cancer mice model by subcutaneously injecting c-Myc overexpression LNCaP. Subsequently, mice were treated with Abi combing Mycro-3. Intriguingly, the single agent Abi treatment exhibited limited outcome, which might be associated with the drugs resistance induced by c-Myc. However, the combination of Mycro-3 and Abi efficiently inhibited the tumor growth (Fig. 5E) and improved the overall survival in mice (Fig. 5F). Those results suggested that interrupt of Trp metabolism/c-Myc loop by c-Myc inhibitor could strengthen the anticancer effects of the chemotherapy, providing an innovative approach in clinical prostatic cancer therapy.

## Discussion

Chemotherapy and radiotherapy are the principal strategies for systemically metastasized carcinomas. Unfortunately, clinical data provides evidence that metastatic prostatic cancer is frequently resistant to diverse antineoplastic agents [26–28]. Current findings have reported extensive metabolic crosstalk between tumor cells and microenvironment, leading to the occurrence of chemoresistance. Thus, there might be potential opportunities to interrupt the aberrant metabolism to improve anticancer effects of current chemotherapy. In fact, Trp catabolism of tumor cells is increasingly being recognized as an essential participant in regulating tumor progression [29]. A newly study provided evidence that activation of Kyn associated signaling pathway might efficiently promote the tumor progression and mortality in prostatic cancer [30]. However, the potential correlation between Trp metabolism and prostatic cancer chemoresistance remained poorly understood. Here, our study firstly pointed out the effect of Trp metabolite on chemoresistance of prostatic cancer and clarified the underlying mechanism.

TDO2 is widely expressed in hepatic tissue, and frequently expressed in other tissues, such as testis, pregnant uterus or brain tissues in particular situation [31, 32]. As for cancer cell lines, over 16% of tumor cell lines revealed certain expression of IDO1, and 19% of those cells are TDO positive [33], reminding us that TDO2 might serve as superior target for precise treatment of cancer. Therefore, we explored whether TDO2 plays a big part in drug-resistant prostatic cancer tissues and cells. Our results indicated a positive correlation of the TDO2 with Kyn in prostatic cancer cells and tumors of drug resistance. Moreover, we demonstrated that C-R tissues could increase the expression of Kyn, upregulate AhR (an endogenous ligand of Kyn [34]), which was dependent on TDO2 and AhR for anchorage apoptosis potential, we speculated that prostatic cancer cells utilized an autocrine signaling loop in which Kyn supported Abi and Dox resistance through an AhR dependent pathway, and the upregulation of TDO2 was central to this signaling loop. The observation that AhR inhibition increased the apoptosis rate of LNCaP and VCaP cells in vitro provided evidence that AhR inhibition represents a novel strategy for targeted therapy for prostatic cancer treatment.

Studies have proved that activation of AhR signals is determined by nuclear export and subsequent AhR degradation in a ubiquitin–proteasome dependent pathway [35]. Apart from the canonical pathway, AhR signals could also be induced by the stimulation from other regulatory proteins. In fact, AhR has been report to interacts with diverse signaling molecules and participated in the activation of cytosolic proteins, including PI3K/AKT, Smads, MPAK and ERK [36]. Interestingly, our results also show that AhR could collaborate with NF-κB to facilitate the activation of c-Myc, and c-Myc overexpression could up-regulate the expression of Trp transporters and ABC transporters, which further elevated the apoptosis rate of Abi or Doc treated prostatic cancer cells. c-Myc, a well-known oncogene, has been reported to cooperate with family members MYCN and MYCL to promote the tumorigenesis of prostatic cancer. Consistently, compelling findings have suggested elevated expression of c-Myc in prostatic cancer tissues [37], which strictly correlates with increased disease severity [38]. Metabolic deregulation is central to the etiology of prostatic cancer [39]; overexpression of c-Myc in tumors has a profound impact on cell metabolism because it induces a global metabolic reprograming that supports cancer cell survival and growth [40]. Recently, a publication provided a novel sight for breast cancer treatment that KPNA2 and IL-6 associated inflammation could facilitate c-Myc nuclear translocation and regulate breast cancer development [41], and this similar mechanism has also been verified in our prostatic cancer cells. More importantly, our finding on how c-Myc overexpression impacts the multidrug resistance of prostatic cancer emphasizes the demand to suppress Trp metabolism in c-Myc associated drugs resistance.

Additionally, the strategy targeting c-Myc signaling loop to overcome chemoresistance still remains a challenge, due to the poor understanding of signaling pathways responsible for chemoresistance in prostatic cancer. Here, we found that c-Myc positively regulates ABC transporters, such as ABCG2, in Abi or Doc-resistant prostatic tumors. Consistent with these findings, the abundance of P-gp in c-Myc overexpression cells was significantly higher compared with the control group. Those results supported our combinatorial strategy, in which combination of c-Myc signaling inhibitor and chemotherapy was conducted to treat prostatic cancer. As a result, the combination exhibited dramatic anticancer effects in our tumor bearing mice.

In this study, we clarified that the inhibition of c-Myc efficiently reduce the tumor growth, and that Abi or Doc combined with Mycro3 had a more exhaustive inhibitory effect on tumor growth than individual treatment. Similarly, either Doc or Abi treatment just slightly prolong the survival of xenograft recipient animals, whereas combining treatment obviously extended the life- span of tumor bearing mice. Thus, the concurrent inhibition of c-Myc and Abi or Doc may be a more effective strategy than the suppression of Abi or Doc alone in Prostatic cancer therapy and could especially be beneficial for patients who have become resistant to chemical-drug. However, it is noteworthy that the mice model with c-Myc overexpression manifested a better tumor growth as well as poor percent survival in Abi treatment than Mycro3, indicating that the overexpression of c-Myc produced drug-resistant in a certain degree. In summary, our results suggest that concurrent treatment of c-Myc signaling inhibitor and chemotherapy may exhibit improved tumor suppressive effects in multidrug-resistant prostatic cancer.

In conclusion, our study demonstrates that elevated TOD2 expression promoted Trp metabolism and metabolite Kyn production, thus resulting in the activation of AhR/c-Myc/ABC-SLC transporters signaling pathway. Interrupt of Trp metabolism/c-Myc loop efficiently suppressed the drugs resistance induced by TDO2, which represented potential target to improve the outcome in drug-resistant prostatic cancer treatment.

## Supplementary Information

**Additional file 1.**

## Data Availability

The datasets used and/or analyzed during the current study are available from the corresponding author on reasonable request.

## Abbreviations

Trp: tryptophan
TDO2: tryptophan 2, 3-dioxygenase 2
Kyn: kynurenine
AhR: aryl hydrocarbon receptor
ABC: ATP-binding cassette
IDO1/2: indoleamine 2,3-dioxygenase-1/2
