# Supplementary figures and images for: Tryptophan metabolism induced by TDO2 promotes prostatic cancer chemotherapy resistance in a AhR/c-Myc dependent manner
Source: BMC Cancer. 2021 Oct 17;21:1112. doi: 10.1186/s12885-021-08855-9 (PMC8520630; doi:10.1186/s12885-021-08855-9)

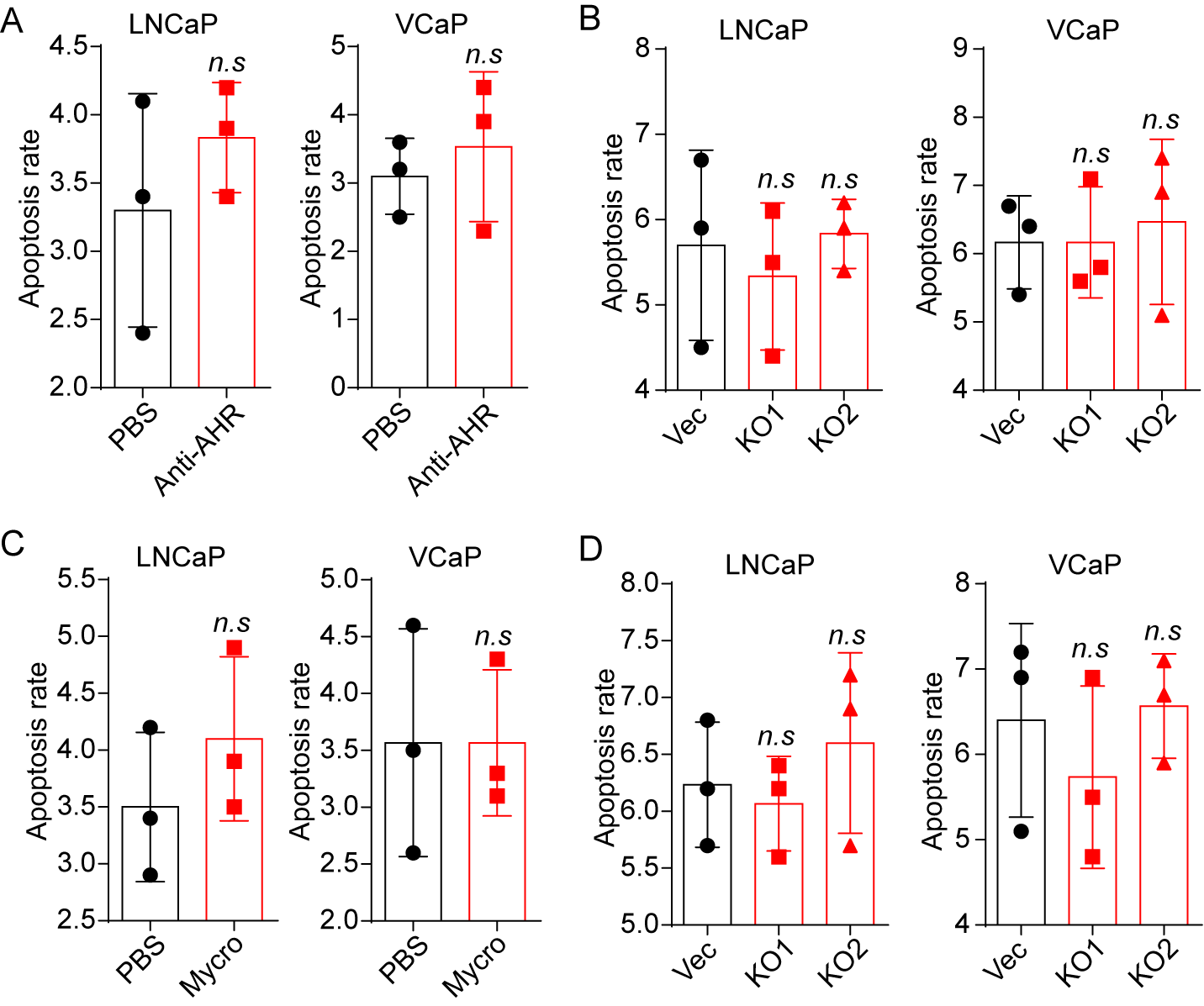

Supplement: Supplementary file 1 — Additional file 1. [file 12885_2021_8855_MOESM1_ESM.tif]
